# Factors associated with false-positive mammography at first screen in an Asian population

**DOI:** 10.1371/journal.pone.0213615

**Published:** 2019-03-11

**Authors:** Peh Joo Ho, Chek Mei Bok, Hanis Mariyah Mohd Ishak, Li Yan Lim, Jenny Liu, Fuh Yong Wong, Kee Seng Chia, Min-Han Tan, Wen Yee Chay, Mikael Hartman, Jingmei Li

**Affiliations:** 1 Genome Institute of Singapore, Genome, Singapore, Singapore, Singapore; 2 School of Biological Sciences, Nanyang Technological University, Singapore, Singapore; 3 Department of Surgery, University Surgical Cluster, National University Hospital, Singapore, Singapore; 4 Saw Swee Hock School of Public Health, National University of Singapore, National University Health System, Singapore, Singapore; 5 National Cancer Centre, Singapore, Singapore; 6 Institute of Bioengineering and Nanotechnology, Singapore, Singapore; 7 Karolinska Institutet, Department of Medical Epidemiology and Biostatistics, Stockholm, Sweden; Nofer Institute of Occupational Medicine, POLAND

## Abstract

**Introduction:**

False-positive recall is an issue in national screening programmes. The aim of this study is to investigate the recall rate at first screen and to identify potential predictors of false-positive recall in a multi-ethnic Asian population-based breast cancer screening programme.

**Methods:**

Women aged 50–64 years attending screening mammography for the first time (*n* = 25,318) were included in this study. The associations between potential predictors (sociodemographic, lifestyle and reproductive) and false-positive recall were evaluated using multivariable logistic regression models.

**Results:**

The recall rate was 7.6% (*n* = 1,923), of which with 93.8% were false-positive. Factors independently associated with higher false-positive recall included Indian ethnicity (odds ratio [95% confidence interval]: 1.52 [1.25 to 1.84]), premenopause (1.23 [1.04 to 1.44]), nulliparity (1.85 [1.57 to 2.17]), recent breast symptoms (1.72 [1.31 to 2.23]) and history of breast lump excision (1.87 [1.53 to 2.26]). Factors associated with lower risk of false-positive recall included older age at screen (0.84 [0.73 to 0.97]) and use of oral contraceptives (0.87 [0.78 to 0.97]). After further adjustment of percent mammographic density, associations with older age at screening (0.97 [0.84 to 1.11]) and menopausal status (1.12 [0.95 to 1.32]) were attenuated and no longer significant.

**Conclusion:**

For every breast cancer identified, 15 women without cancer were subjected to further testing. Efforts to educate Asian women on what it means to be recalled will be useful in reducing unnecessary stress and anxiety.

## Introduction

Screening mammography is a low-dose X-ray-based imaging tool used in the early detection of breast cancer before symptoms appear. It is the main breast cancer screening tool known to reduce deaths from the disease [1]. The reduction in breast cancer mortality rates attributed to the success of mammography screening is estimated to be between 25–40% [2, 3]. However, screening is not without consequences. If abnormalities were found on a mammogram, a woman is often recalled and referred to an assessment clinic for additional imaging and follow-up tests (which may be invasive). While higher sensitivity (proportion of true-positive results, or tests that correctly indicate a woman has breast cancer) is sought after, this results in higher recall rate and inevitably leads to increased number of false-positive recalls [4].

False-positive recalls are undesirable as they incur unnecessary direct out-of-pocket costs, and are often associated with other indirect and intangible harms [5]. The necessary follow-up mammography examinations increase the exposure of women to more ionizing radiation that may increase breast cancer risk [6]. Recall after mammography among women with a false-positive mammogram can provoke anxiety (persisting for one to three years) and lead to depression [7–9]. The level of distress experienced from false-positive recall was found to be no different from that of a breast cancer diagnosis for six months after a recall episode [9]. Several studies showed a lower screening re-attendance rate after a false-positive recall, which has a negative impact on the overall success of a screening program [10–15].

Demographic and lifestyle factors associated with breast cancer risk could be used to improve the screening strategy to reduce false-positive recall [16–18]. Younger age, African-American descent, lower body mass index, premenopausal, no children, and the use of hormone replacement therapy were found to be associated with higher likelihood of false-positive recall [16, 17, 19–22]. In addition, positive associations between false-positive recall and other breast cancer risk factors such as family history of breast cancer, history of benign breast disease and mammographic density have been reported [16–18]. However, differences between health systems in different countries and characteristics of the populations make it difficult to generalize results from predominantly White populations to Asian women [23].

In Singapore, it is recommended that women aged 50 years and above go for routine mammography screening every two years [24]. Although more women here are being diagnosed with breast cancer each year, only 66% of the main target group of women aged 50 to 69 ever had a mammogram, and half of them do not come back for regular screening at two-year intervals, thus negating the benefit of a nationwide mammography screening program (Health Promotion Board, Singapore). One of the possible reasons for the low uptake of follow-up screening could due to the negative emotional impact from false-positive recall. Using data from a multi-ethnic population-based breast cancer screening project we investigated the recall rate at first screen and studied potential predictors of false-positive recall in Singapore.

## Methods

### Study population

**Fig 1:**
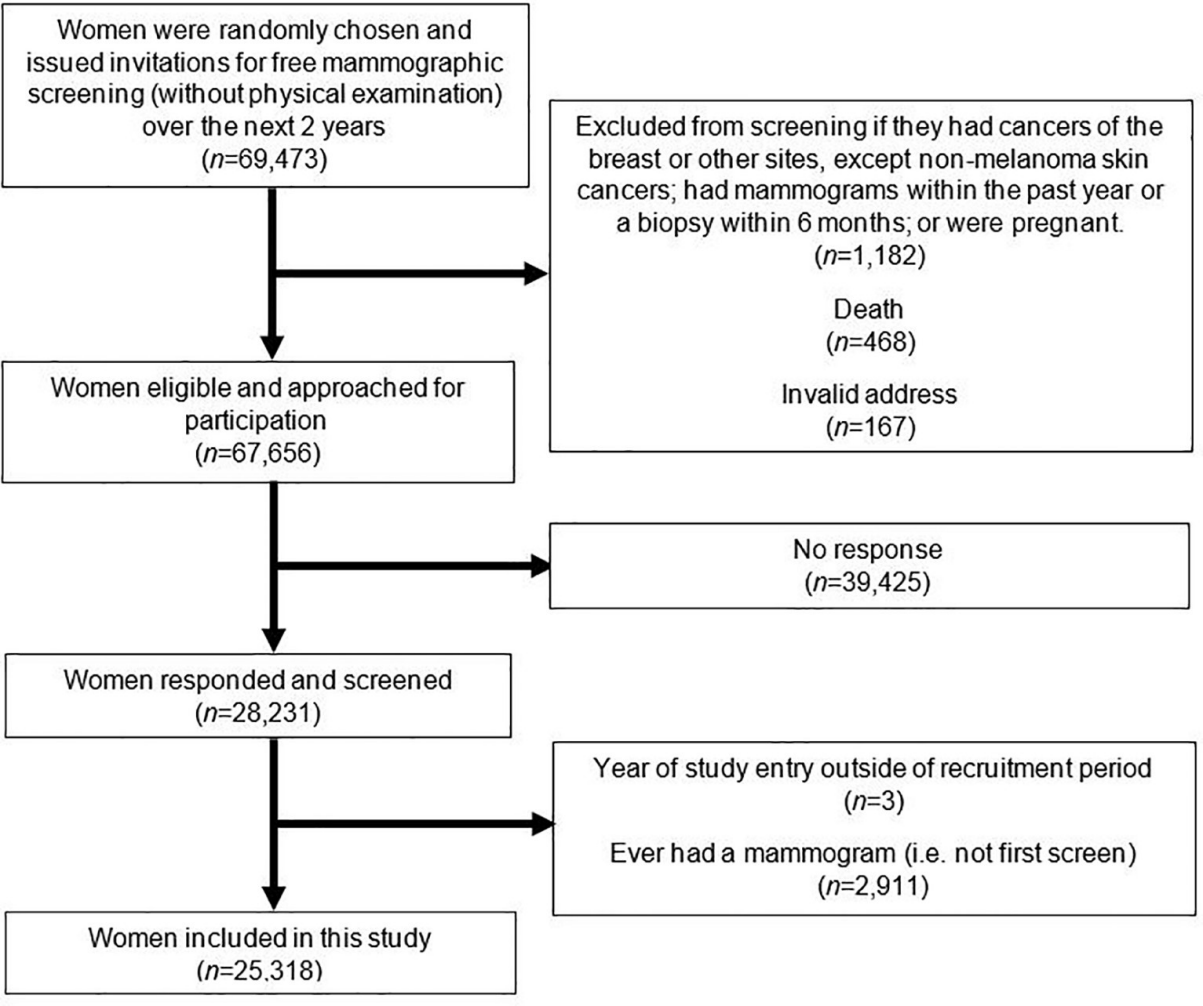
Flow diagram of how analytical cohort was derived.

The Singapore Breast Cancer Screening Project (SBCSP) was a population-based prospective trial of screening mammography in Singapore. The details of the program have been previously described [25]. Briefly, 69,473 women aged 50–64 years were randomly selected and invited for a single two-view mammogram examination from 1994 through 1997. Screening was conducted at two sites, Singapore General Hospital (SGH) and Toa Payoh Hospital (TPH). Women were excluded if they had cancers of the breast or other sites (except non-melanoma skin cancer), had mammography done or breast biopsy in the past one year prior to screening, or were pregnant (*n* = 1,182). Further exclusions were made due to death (*n* = 468) or invalid address (*n* = 167). Of the remaining 67,656 women, 28,231 (41.7%) participated and were screened as part of SBCSP (see flow diagram in **Fig 1**).

In this study, we further excluded 2,911 women who have had mammography done within 2 years prior to the screening project (i.e. not first screen). In addition, three women with years of study entry outside of the recruitment period were excluded, resulting in a final analytical dataset of 25,318 women. This study uses existing anonymous data from the Singapore Breast Cancer Screening Project. The original study received ethics approval from SingHealth Centralised Institutional Review Board (REF: 205–001).

### Identification of breast cancer cases

Invasive and non-invasive breast cancer cases diagnosed within one year after study entry (date of screen) were identified either through SBCSP or via record linkage with the Singapore Cancer Registry using national registration identity card numbers unique to each subject. The population-based national registry records all cases of cancer diagnosed in Singapore [26].

### Definition of recall and false-positive recall

“Recall rate” is defined as the proportion of screening mammography examinations resulting in a recommendation for further workup (either a further radiologic assessment or a joint assessment) [4]. Women who were recommended for recall were considered to have a positive screening mammogram. A positive mammogram was further classified as false-positive if no breast cancer (invasive or non-invasive) was diagnosed within a year of the screening examination [27].

### Variables of interest

Participants of the SBCSP were asked to complete a questionnaire covering major breast cancer risk factors related to background and lifestyle (age at study entry/mammography screen, screening site, ethnicity, education, body mass index, smoking status, family history of breast cancer, Pap smear attendance), hormonal and reproductive characteristics (age at menarche, menopausal status, number of children, age at first birth, breastfeeding history, oral contraceptive use, hormone replacement therapy), and breast health (breast symptoms [i.e. pain/tenderness, lump, thickening, nipple eczema, nipple discharge, nipple inversion, or skin change] in past one month, ever had breast lump biopsied) [28].

### Features of lesions

Descriptions of radiologic features were obtained from mammography reports completed by two radiologists who were blinded to each other’s interpretations. Notes on parenchyma appearance (normal, dense or fatty) and film quality (good, medium or poor) were available for all mammograms. For mammograms on which a lesion was found, the presence or absence of the following features were noted by the attending radiologist: mass, outline (smooth/irregular), microcalcification, stromal distortion, asymmetric increase in density, skin involvement of nipple retraction, lymph nodes, and classification (benign, equivocal or malignant). In cases where two mammogram reports were available, the more severe phenotype was taken.

### Assessment of mammographic density

As described previously in Lee *et al*., screen-film mammography images were collected and digitized between February 2012 through February 2013 with the Array 2905HD Laser Film Digitizer (Array Corp, Tokyo, Japan) at a sampling pitch of 50 micrometers and a gray-scale contrast resolution of 12 bits [29]. Percent mammographic density of mammograms in the mediolateral oblique view was measured using a fully-automated thresholding method previously described [29, 30]. Briefly, the algorithm uses a machine learning approach to mimic percent mammographic measurements made using Cumulus, the gold standard method for measuring mammographic density. The mean density of both breasts was calculated.

### Statistical analysis

Frequencies and relative percentages were reported for categorical variables for total number of recalled mammograms and for false-positive mammograms. The Chi-square test was used to test the association of having a recalled mammogram (or false-positive mammogram) with categorical variables of interest. The effect sizes of the associations between each factor and false-positive recall were estimated using logistic regression models adjusting for screening site. Multivariable logistic regression models were fitted to study factors independently associated with false-positive recall at first screen. The variables included screening site and all factors found to be significantly associated with having false-positive recalled mammograms using the Chi-square tests. All statistical analyses were performed using R (version 3.4.3).

## Results

Of the 25,318 women included in this study, 1,923 were recalled for assessment (recall rate = 7.6%). Cancer detection rate (*n* = 124) was ~0.5%. Less than 1 in 16 of the women recalled were found to have a breast cancer detected during screening (6.4% of all recalled).

**Fig 2:**
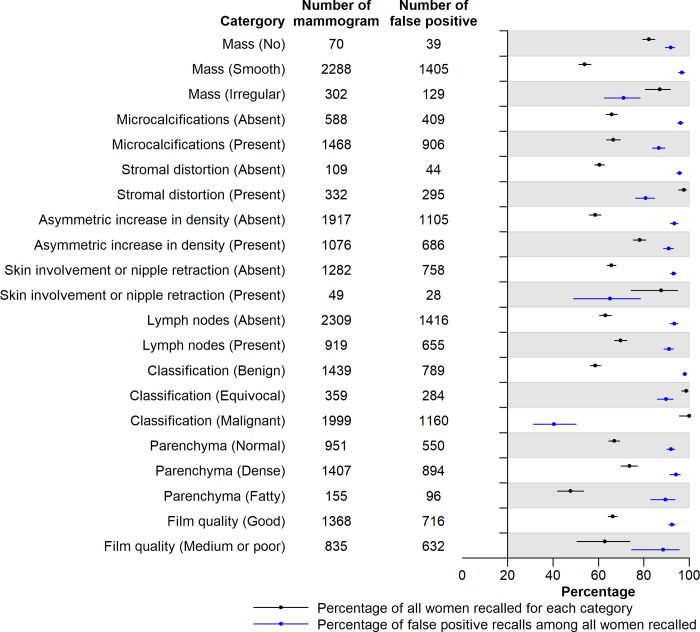
Characteristics of lesions found in 2,358 women in the Singapore Breast Cancer Screening Project. Of the women with reported lesions found on their screening mammograms, 1,562 (66%) were recalled.

From the mammography reports, 2,358 lesions with suspicious mammographic patterns were found. Sixty-six percent of the women with lesions were recalled for further testing. Women most frequently recalled showed tumor-like masses (classified malignant [100% recalled, 95.8 to 100], classified equivocal [98.8%, 96.7 to 99.6], irregular outline [87.1%, 80.5 to 91.8]), stromal distortion (97.8%, 95.5 to 99.0), skin involvement or nipple retraction (87.8%, 74.5 to 94.9), asymmetric densities (78.2%, 75.4 to 80.8), dense parenchymal tissue (73.8%, 70.0 to 77.3), suspicious axillary lymph nodes (69.9%, 67.0 to 72.6) and presence of microcalcifications (66.7%, 63.6 to 69.6) (**Fig 2**). Among the recalled mammograms, false-positive recall rate were highest for abnormalities related to equivocal masses (89.9%, 86.0 to 92.9), stromal distortion (80.9%, 76.3 to 84.8), asymmetric densities (91.1%, 88.7 to 93.0), and dense parenchymal tissue (94.2%, 91.5 to 96.2) (**Fig 2**).

**Table 1** shows the percentage of overall recall and false-positive recall by various factors. The proportion of women who received false-positive results varied significantly by age, ethnicity, education, body mass index (BMI), family history of breast cancer, age at menarche, menopausal status, parity, ever breastfed, oral contraceptive use and variables related to breast health (breast symptoms, ever had breast lump removed or biopsied and percent mammographic density). No significant difference was found for screening site, regular smoking, age at first birth, hormone replacement therapy, Pap smear attendance and clinical breast examination.

**Table 1:** Descriptive characteristics of Singapore Breast Cancer Screening Project. Pvalues from Chi-Square test comparing characteristics observed in women recalled and women not recalled (P_1_) and in women with false positive recall and women not recalled (P_2_) were reported.

Variable	*n*	Number recalled (*n*,%)	P_1_	False positive (*n*,%)	P_2_
**Background and lifestyle**					
Age at study entry, years					
<55	7,301	591 (8.1)	<0.001	563 (7.7)	<0.001
55–59	9,378	766 (8.2)		713 (7.6)	
≥60	8,639	566 (6.6)		523 (6.1)	
Site					
SGH	21,685	1,625 (7.5)	0.145	1,520 (7.0)	0.156
TPH	3,633	298 (8.2)		279 (7.7)	
Ethnicity					
Chinese	21,201	1,590 (7.5)	<0.001	1,479 (7.0)	<0.001
Malay	1,533	95 (6.2)		90 (5.9)	
Indian	1,268	133 (10.5)		128 (10.1)	
Others	1,316	105 (8.0)		102 (7.8)	
Education					
No formal education	16,385	1,194 (7.3)	0.013	1,114 (6.8)	0.011
Formal education (≥6 years)	8,933	729 (8.2)		685 (7.7)	
Body mass index, kg/m^2^					
≥25	11,299	813 (7.2)	0.033	760 (6.7)	0.037
<25	14,008	1,109 (7.9)		1,038 (7.4)	
Regular smoking					
No	24,260	1,855 (7.6)	0.160	1,736 (7.2)	0.153
Yes	1,058	68 (6.4)		63 (6.0)	
Family history of breast cancer					
No	24,458	1,837 (7.5)	0.019	1,718 (7.0)	0.027
Yes	567	58 (10.2)		54 (9.5)	
Ever attended Pap smear					
Yes	15,385	1,132 (7.4)	0.078	1,063 (6.9)	0.135
No	9,903	789 (8.0)		734 (7.4)	
**Hormonal and reproductive**					
Age at menarche, years					
≥14	1,6671	1,214 (7.3)	0.011	1,138 (6.8)	0.019
<14	8,636	707 (8.2)		659 (7.6)	
Menopause status					
Post	2,2831	1,692 (7.4)	0.001	1,581 (6.9)	0.001
Pre	2,474	229 (9.3)		216 (8.7)	
Number of children					
≥3	19,800	1,364 (6.9)	<0.001	1,280 (6.5)	<0.001
1–2	3,707	315 (8.5)		298 (8.0)	
0	1,811	244 (13.5)		221 (12.2)	
Age at first birth*					
≥25	9,056	664 (7.3)	0.557	617 (6.8)	0.778
20–24	9,703	672 (6.9)		636 (6.6)	
<20	4,596	326 (7.1)		308 (6.7)	
Ever breastfed*					
Yes	16,410	1,122 (6.8)	0.012	1,061 (6.5)	0.043
No	6,946	541 (7.8)		501 (7.2)	
Oral contraceptive use					
No	15,806	1,290 (8.2)	<0.001	1,205 (7.6)	<0.001
Yes	9512	633 (6.7)		594 (6.2)	
Hormone replacement therapy					
No	22,652	1,697 (7.5)	0.075	1,586 (7.0)	0.066
Yes	2,666	226 (8.5)		213 (8.0)	
**Breast health**					
Breast symptoms in past one month					
No	24,790	1,834 (7.4)	<0.001	1,732 (7.0)	<0.001
Yes	527	89 (16.9)		67 (12.7)	
Ever had breast lump biopsied					
No	24,332	1,785 (7.3)	<0.001	1,669 (6.9)	<0.001
Yes	986	138 (14.0)		130 (13.2)	
Ever had clinical breast examination					
Yes	12,996	988 (7.6)	0.985	927 (7.1)	0.881
No	12,322	935 (7.6)		872 (7.1)	
Percent mammographic density					
<10	3,705	121 (3.3)	<0.001	117 (3.2)	<0.001
10–24	12,722	861 (6.8)		818 (6.4)	
≥25	5,844	643 (11.0)		593 (10.1)	

*For a subset of women who have children.

Factors associated with higher false-positive recall after adjusting for screening site were Indian ethnicity (1.50 [1.23 to 1.80]), formal education (1.13 [1.03 to 1.25]), lower BMI (1.11 [1.01 to 1.22]), family history of breast cancer (1.39 [1.04 to 1.83]), below median age at menarche (1.12 [1.02 to 1.24]), premenopause (1.29 [1.11 to 1.49]), no children (2.03 [1.74 to 2.36]), self-reported breast symptoms in the past month (2.02 [1.54 to 2.60]), history of biopsied breast lump (2.07 [1.70 to 2.50]) and higher percent mammographic density (3.48 [2.85 to 4.28]) (**Table 2**). Factors associated with lower false-positive recall after adjusting for screening site were older age (0.77 [0.68 to 0.87]) and use of oral contraceptives (0.81 [0.73 to 0.89]) (**Table 2**).

**Table 2:** Predictors of false-positive mammography recall at first screen, adjusted for screening site (odds ratio [OR] and 95% confidence intervals [CI]). Bold indicates significant associations at p<0.05.

	Univariate	Adjusted for site
Variable	OR (95% CI)	OR (95% CI)
**Background and lifestyle**		
**Site**		
** SGH**	1.00 (Reference)	
** TPH**	1.10 (0.97 to 1.26)	-
Age at study entry, years		
<55	1.00 (Reference)	1.00 (Reference)
55–59	0.99 (0.88 to 1.11)	0.99 (0.88 to 1.11)
≥60	**0.77 (0.68 to 0.87)**	**0.77 (0.68 to 0.87)**
Ethnicity		
Chinese	1.00 (Reference)	1.00 (Reference)
Malay	0.83 (0.66 to 1.03)	0.84 (0.67 to 1.03)
Indian	**1.50 (1.23 to 1.80)**	**1.50 (1.23 to 1.80)**
Others	1.12 (0.90 to 1.37)	1.12 (0.91 to 1.38)
Education		
No formal education	1.00 (Reference)	1.00 (Reference)
Formal education (≥6 years)	**1.14 (1.03 to 1.26)**	**1.13 (1.03 to 1.25)**
Body mass index, kg/m^2^		
≥25	1.00 (Reference)	1.00 (Reference)
<25	**0.90 (0.82 to 0.99)**	**0.90 (0.82 to 0.99)**
Regular smoking		
No	1.00 (Reference)	1.00 (Reference)
Yes	0.82 (0.63 to 1.06)	0.82 (0.63 to 1.06)
Family history of breast cancer		
No	1.00 (Reference)	1.00 (Reference)
Yes	**1.40 (1.04 to 1.84)**	**1.39 (1.04 to 1.83)**
Ever attended Pap smear		
Yes	1.00 (Reference)	1.00 (Reference)
No	1.08 (0.98 to 1.19)	1.08 (0.98 to 1.19)
**Hormonal and reproductive**		
Age at menarche, years		
≥14	1.00 (Reference)	1.00 (Reference)
<14	**1.13 (1.02 to 1.25)**	**1.12 (1.02 to 1.24)**
Menopause status		
Post	1.00 (Reference)	1.00 (Reference)
Pre	**1.29 (1.11 to 1.49)**	**1.29 (1.11 to 1.49)**
Number of children		
≥3	1.00 (Reference)	1.00 (Reference)
1–2	**1.27 (1.11 to 1.44)**	**1.26 (1.10 to 1.44)**
0	**2.03 (1.74 to 2.36)**	**2.03 (1.74 to 2.36)**
Age at first birth*		
≥25	1.00 (Reference)	1.00 (Reference)
20–24	0.96 (0.85 to 1.07)	0.96 (0.86 to 1.08)
<20	0.98 (0.85 to 1.13)	0.98 (0.85 to 1.13)
Ever breastfed*		
Yes	1.00 (Reference)	1.00 (Reference)
No	**1.13 (1.01 to 1.26)**	**1.12 (1.00 to 1.25)**
Oral contraceptive use		
No	1.00 (Reference)	1.00 (Reference)
Yes	0.81 (0.73 to 0.89)	**0.81 (0.73 to 0.89)**
Hormone replacement therapy		
No	1.00 (Reference)	1.00 (Reference)
Yes	1.15 (0.99 to 1.34)	1.15 (0.99 to 1.34)
**Breast health**		
Breast symptoms in past one month		
No	1.00 (Reference)	1.00 (Reference)
Yes	**2.03 (1.55 to 2.61)**	**2.02 (1.54 to 2.60)**
Ever had breast lump biopsied		
No	1.00 (Reference)	1.00 (Reference)
Yes	**2.07 (1.70 to 2.50)**	**2.07 (1.70 to 2.50)**
Ever had clinical breast examination		
Yes	1.00 (Reference)	1.00 (Reference)
No	0.99 (0.90 to 1.09)	0.99 (0.90 to 1.09)
Percent mammographic density		
<10	1.00 (Reference)	1.00 (Reference)
10–24	**2.11 (1.74 to 2.59)**	**2.11 (1.74 to 2.58)**
≥25	**3.49 (2.86 to 4.30)**	**3.48 (2.85 to 4.28)**

*For a subset of women who have children.

**Fig 3:**
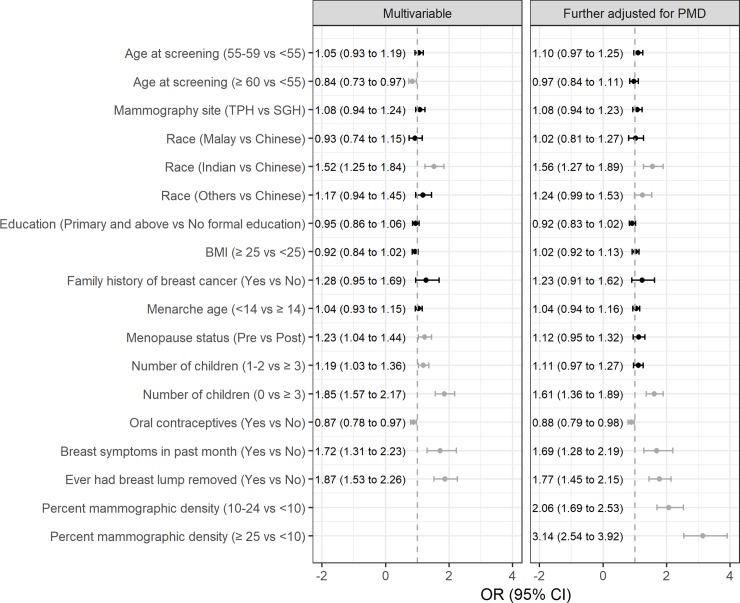
Factors associated with false-positive mammography recall at first screen. Results associated with P<0.05 are shown in dark grey. OR: odds ratio; 95% CI: 95% confidence intervals.

Younger age at screen (≥60 vs. <50 years: 0.84 [0.73 to 0.97]), Indian ethnicity (Indian vs. Chinese: 1.52 [1.25 to 1.84]), premenopause (pre vs. post: 1.23 [1.04 to 1.44]), no children (0 vs. ≥3: 1.85 [1.57 to 2.17]), never used of oral contraceptives (ever use vs. never: 0.87 [0.78 to 0.97]), recent breast symptoms (yes vs. no: 1.72 [1.31 to 2.23]) and history of breast lump excision (yes vs. no: 1.87 [1.53 to 2.26]) were independently associated with higher likelihood of false-positive recall (**Fig 3**). After further adjusting for percent mammographic density measured from the screening mammogram, the associations of older age at screening and premenopausal status with false-positive recall were attenuated (0.97 [0.84 to 1.11] and 1.12 [0.95 to 1.32], respectively) (**Fig 3**).

**Fig 4:**
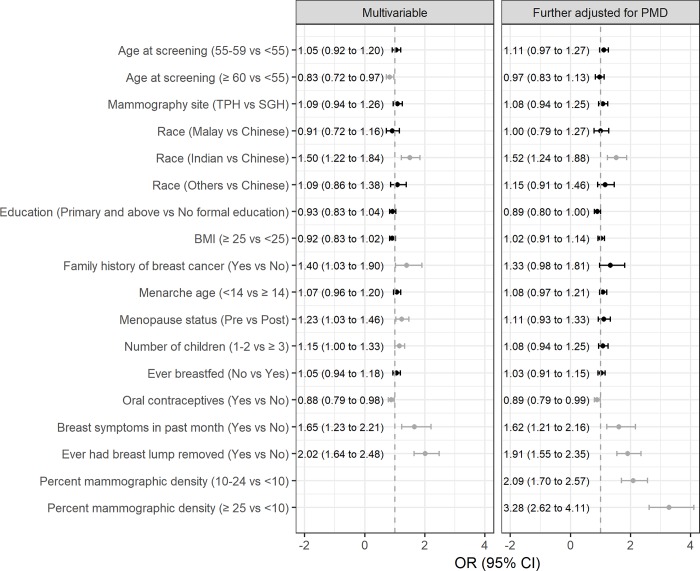
Factors associated with false-positive mammography recall at first screen in a subset of women with children. Results associated with P<0.05 are shown in dark grey. OR: odds ratio; 95% CI: 95% confidence interval.

In a subset of women who have children, women who have never breastfed were associated with a higher likelihood of false-positive recall (1.12 [1.00 to 1.25]) (**Table 2**). However, breastfeeding was not found to be an independent predictor of false-positive recall (**Fig 4**).

The results (**Figs 3** and **4**) were not appreciably different when we limited the analysis to women without breast symptoms. The effect of statistically significant variables were not appreciably changed upon the exclusion of non-significant variables in models from **Figs 3** and **4**.

## Discussion

We observed a recall rate of 7.6%, of which 93.6% of the recalls were false-positive. Ethnicity, number of children, oral contraceptive use, recent breast symptom, history of breast lump excision and percent mammographic were identified to be independent predictors of false-positive mammography recall at first screen. Our findings for the variables (i.e. cigarette smoking, family history of breast cancer, and education) that did not show significant association with false-positive recall were supported by previous literature [16].

The high rate of false-positive recall observed is not surprising, given that common benign breast disease and breast cancer may present with similar radiologic features on abnormal mammogram. Nicola *et al*. [31] reported that 55% of biopsies performed on the basis of mammographic changes, for instance microcalcification, stromal distortion and spiculation, are due to benign breast disease. Another study has shown that 33% of the biopsies done were due to benign lesions [32]. Most women who complain of breast pain or palpable masses are diagnosed with benign breast disease [33, 34]. In concordance, we observed that false-positive recalls were more common among women who reported breast symptoms (pain/tenderness, or breast lump, or other breast complaints) for the past month before the screen. Furthermore, the risk of false-positive recall is also increased among women with history of breast lump excision, which can result in tissue distortion and scaring [16, 17, 22, 35, 36].

Higher mammogram density may obscure subtle signs of malignancy and thus the woman may be recalled more frequently or be subjected to additional mammographic views [28]. McGuinness *et*. *al*. found that having a high breast density is significantly associated with having a false-positive recall [18]. In our study, the associations between increased risk of false-positive recall with age at screening and menopausal status were attenuated after adjusting for breast density. This is in agreement with Martin *et*. *al*., whereby density of breast tissue was reported to be inversely correlated with body size, age and menopause [37]. In addition, the association between nulliparity and an increased risk for false-positive recall observed in this study is consistent with findings presented by Banks *et al*. [16]. Women without children are known to have higher mammographic density than parous women in general, which may in turn lead to an increased risk of false-positive recall [38, 39].

Previous literature have shown that when compared to other ethnic groups (White, African-American, Hispanic and others), older Asian women experienced the lowest false-positive rate, followed by African-Americans and then the white population [17]. Asians are a heterogeneous group, with a paucity of literature on false-positive recall rates by ethnicity. It has been previously shown that breast cancer risk and percent mammographic density were highest amongst Chinese women, compared to Indians and Malays [40, 41]. Intuitively, the likelihood of false-positive recall would be highest among the Chinese, who on average, have higher mammographic density (dense tissue makes it more difficult to evaluate abnormalities on a mammogram). On the contrary, we found that ethnic Indian women in our screening population showed a higher risk for false-positive recall as compared to Chinese, despite accounting for the differences in percent mammogram density. A likely explanation for the higher false-positive recall rate could be due to the higher incidence rate of fibroadenoma (i.e. a type of benign breast disease) among Indian women [42].

We found that the use of oral contraceptives is significantly associated with the risk of false-positive recall. Tomasson *et al*. observed a protective, although not statistically significant, effect of ever use of oral contraceptive on the development of breast cancer in Icelandic women (1–48 months vs no use: 0.92 [0.73 to 1.16]) [43]. In a subgroup of women who were diagnosed with cancer under 45 years, the use of oral contraceptives did have an appreciable protective effect against breast cancer (1–48 months vs no use: 0.64 [0.44 to 0.94]) [43]. They speculated that oral contraceptives high in estrogen content increased the risk of breast cancer and that progestogen-only oral contraceptives was protective [43]. In contrast, in a meta-analysis by Nelson *et*. *al*. involving studies of any oral contraceptive use (combination, progestin- and estrogen-only formulations), no significant association was found with the risk of false-positive recall [16, 17].

The main strengths of the study include the large sample of women aged 50–60 in Singapore and the overall completeness of 98.1% of the Singapore Cancer Registry [44]. In addition, data from the mammography reports on the lesions and mass were available to be studied. Our study is not without limitations. Limitations associated with the study include potential selection bias. While a random sample of women was invited to participate in the screening program, the low response rate may have resulted in a sample of women who were more health conscious or of higher education. This may limit the generalization of our results to the whole of Singapore. In addition the period of time the data represents, i.e. 1994–1997, the effect sizes of the predictors of false positive mammogram in the current screening setting may differ. Film mammography has progressively been replaced by digital mammography in recent years. While the improved resolution may reduce the number of recalls with experience, the associations between the potential predictors and false-positive recall are likely to remain [45]. The lifestyle factors of the current screening population may show different trends from those in the 1990s, for example in education level, number of children and age at first birth. More sites (other than SGH and TPH) are also involved in the nationwide mammography programme. The difference between radiologists at the different sites may introduce site difference in false positive results that was not observed in our study. Our study also did not account for radiologists’ interpretive volume (i.e. number of mammograms read), which is believed to contribute in the accuracy of the screening mammography [46].

For every breast cancer identified, 15 women without cancer were subjected to further assessments which may be physically and mentally stressful. Indian ethnicity, nulliparous, never users of oral contraceptive, recent breast symptom, history of breast lump excision and percent mammographic were identified to be independent predictors of having a false-positive mammography at first screen. Efforts to educate Asian women on what it means to be recalled will be useful in reducing unnecessary stress and anxiety. It is important that screening programmes seek to find an optimal balance between recall rate and definitive cancers detected so that resources can be utilized productively.
